# Beneficial effect of STAT3 decoy oligodeoxynucleotide transfection on organ injury and mortality in mice with cecal ligation and puncture-induced sepsis

**DOI:** 10.1038/s41598-020-72136-x

**Published:** 2020-09-17

**Authors:** Samar Imbaby, Naoyuki Matsuda, Kengo Tomita, Kohshi Hattori, Sailesh Palikhe, Hiroki Yokoo, Yuichi Hattori

**Affiliations:** 1 Department of Molecular and Medical Pharmacology, Graduate School of Medicine and Pharmaceutical Sciences, University of Toyama, Toyama, Japan; 2 Department of Emergency and Critical Care Medicine, Nagoya University Graduate School of Medicine, Nagoya, 466-0065 Japan; 3 Department of Anesthesiology and Pain Relief Center, The University of Tokyo Hospital, Tokyo, Japan; 4 Department of Health and Nutritional Sciences, Faculty of Health Promotional Sciences, Tokoha University, Hamamatsu, Japan; 5 Advanced Research Promotion Center, Health Sciences University of Hokkaido, Tobetsu, Japan; 6 Present Address: Department of Clinical Pharmacology, Faculty of Medicine, Suez Canal University, Ismailia, Egypt; 7 Present Address: Institute of Technology, Shimizu Corporation, Tokyo, Japan

**Keywords:** Drug development, Infectious diseases, Molecular medicine

## Abstract

Sepsis is a major clinical challenge with unacceptably high mortality. The signal transducers and activators of transcription (STAT) family of transcription factors is known to activate critical mediators of cytokine responses, and, among this family, STAT3 is implicated to be a key transcription factor in both immunity and inflammatory pathways. We investigated whether in vivo introduction of synthetic double-stranded STAT3 decoy oligodeoxynucleotides (ODNs) can provide benefits for reducing organ injury and mortality in mice with cecal ligation and puncture (CLP)-induced polymicrobial sepsis. We found that STAT3 was rapidly activated in major end-organ tissues following CLP, which was accompanied by activation of the upstream kinase JAK2. Transfection of STAT3 decoy ODNs downregulated pro-inflammatory cytokine/chemokine overproduction in CLP mice. Moreover, STAT3 decoy ODN transfection significantly reduced the increases in tissue mRNAs and proteins of high mobility group box 1 (HMGB1) and strongly suppressed the excessive elevation in serum HMGB1 levels in CLP mice. Finally, STAT3 decoy ODN administration minimized the development of sepsis-driven major end-organ injury and led to a significant survival advantage in mice after CLP. Our results suggest a critical role of STAT3 in the sepsis pathophysiology and the potential usefulness of STAT3 decoy ODNs for sepsis gene therapy.

## Introduction

Despite recent advances in overall medical management, sepsis and its life-threatening complication, septic shock, remain the main cause of mortality in critically ill patients and are present as a public health problem requiring population-based and systems-based solutions^1,2^. The development of dysfunction/failure of one or more organs is a major concern in the sepsis pathophysiology and could pose a major threat to the survival of septic patients^3,4^. Quite recently, the third international consensus definitions have been reviewed for sepsis and septic shock. In short, sepsis has been now redefined as a life-threatening organ dysfunction due to a dysregulated response of the host to infection^5^. However, the pathophysiological process in the development of sepsis-associated multiple organ dysfunction remains incompletely understood, and the lack of effective treatment of septic key organ failure concerns as a major hurdle in its clinical management^6^. Accordingly, the identification for development of therapies aimed at preventing or limiting molecular events associated with the progress of fatal key organ failure, hence leading to improvement of outcomes, would be a pressing issue.

The pathological hallmarks of sepsis derive from the induction of a wide variety of genes and their products. The signaling pathways activated as cellular events during sepsis energize different transcription factors, which coordinate the induction of myriad of genes encoding pathological mediators^7,8^. Nuclear factor-κB (NF-κB) is a family of key transcription factors that are present in virtually all cell types and many of pro-inflammatory genes are regulated by its responsive site, κB, in the DNA^9^. Several prior studies have shown the benefit of pharmacological interventions designed to inhibit activation of NF-κB in rodent models of endotoxic shock^10–13^. In our previous reports, we have introduced in vivo synthetic double-stranded decoy oligodeoxynucleotide (ODN) containing selective NF-κB protein dimer binding consensus sequences into mice with lipopolysaccharide (LPS)- and cecal ligation and puncture (CLP)-induced sepsis^14–16^. In vivo transfection of NF-κB decoy ODNs have been found to result in evident reductions in blood levels of pro-inflammatory cytokines and acute lung injury in septic mice^14–16^. However, NF-κB decoy ODN treatment has failed to confer an effectual survival advantage to mice with CLP-induced sepsis^16,17^. We have also demonstrated that in vivo administration of decoy ODNs with a circular dumbbell structure binding to another transcription factor activator protein-1 (AP-1), which is activated during sepsis in a different time-dependent manner from NF-κB, can lead to a significant survival advantage in the late phase of sepsis in mice rendered septic by CLP without effect on early mortality^18^. This suggests that AP-1 may play a pivotal role as a transcription factor in the late phase of sepsis^17,18^.

The signal transducers and activators of transcription (STAT) family of transcription factors was originally identified through the careful analysis of the molecular requirements of interferon (IFN)-triggered gene expression^19^. Since then, the STAT family is known to be composed of seven members, STAT1, STAT2, STAT3, STAT4, STAT5A, STAT5B, and STAT6, that transduce signal from a variety of extracellular stimuli initiated by different cytokine families^19^. The STAT proteins are present in a latent form in the cytoplasm and become activated through tyrosine phosphorylation which occurs upon activation of the Janus kinase (JAK) family and the Src family^20,21^. Phosphorylated STATs form homo- or hetero-dimers, translocate to the nucleus, and regulate transcription of many target genes^22^. Among the STAT family, STAT3 has been implicated to be a leading transcription factor in both immunity and inflammatory pathways^23,24^. Although mouse strains with a conditional deletion of the STAT3 gene in macrophages/neutrophils or endothelial cells display an increased susceptibility to LPS, leading to increased production of pro-inflammatory cytokines possibly due to defective interleukin (IL)-10-induced STAT3 activation^25,26^, several lines of evidence indicate a critical role of STAT3 tyrosine phosphorylation in production of pro-inflammatory cytokines, including IL-1β and IL-6, in LPS-stimulated macrophages^27–29^. Of note, it has been suggested that STAT3 activation may play a role in mediating acute lung injury after intraperitoneal and intranasal LPS administration in mice^30^. Moreover, the protective effect of STAT3 inhibition on LPS-induced acute lung injury has been demonstrated using the small-molecule STAT3 inhibitor LLL12^31^. However, the pathogenetic role, if any, of the STAT3 pathway in the development of key organ dysfunction in sepsis remains to be explored.

In this study, we introduced in vivo synthetic double-stranded decoy ODN containing selective STAT3 protein dimer binding consensus sequences into mice with CLP-induced sepsis. We also examined how pharmacological intervention using stattic, a small-molecule inhibitor of STAT3 activation and dimerization^32^, can affect the sepsis pathology in CLP mice. Our studies demonstrate that inhibiting the STAT3 pathway can downregulate pro-inflammatory cytokines, reduce tissue inflammation, minimize the development of key organ failure, and improve survival in septic mice, suggesting that STAT3 may be developed as an attractive and potential therapeutic target for treatment of sepsis.

## Results

### STAT3 activation in tissues from mice after CLP and in vivo transfection of STAT3 ODNs

**Figure 1:**
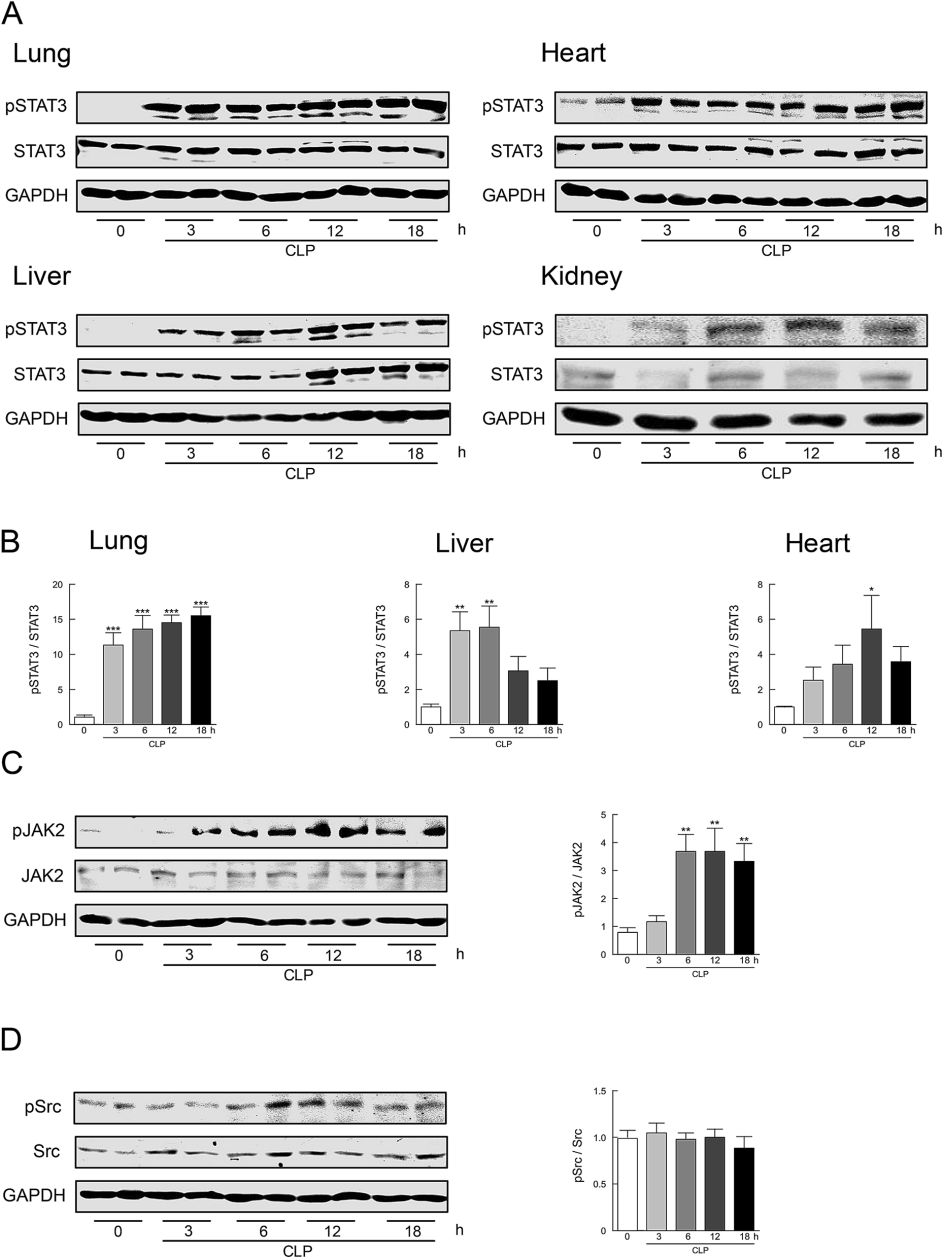
Activation of STAT3 in mouse tissues following CLP-induced sepsis. (**A**) Time course of changes in STAT3 phosphorylation in lung, liver, kidney, and heart tissues after CLP. Representative Western blots of phospho-STAT3 at Tyr-705 and of total STAT3 are shown. (**B**) The summarizing data obtained in lung, liver, and heart tissues are presented as phospho-STAT3/STAT3 expressed relative to the respective control (sham-operated) (*n* = 8). (**C**) Time course of phosphorylation levels of JAK2 at Tyr-1007 and Tyr-1008 in liver tissues were quantified by Western blot analysis (*n* = 8). (**D**) Time course of phosphorylation levels of Src at Tyr-416 in liver tissues were quantified by Western blot analysis (*n* = 8). Typical Western blots are shown in the left side and the summary of quantification of densitometric measurement as a ratio of phosphorylated molecule relative to total expression is presented in the right side of each panel. GAPDH served as loading control. *P < 0.05, **P < 0.01, and ***P < 0.001 compared with sham-operated control (0 h).

We initially ascertained whether STAT3 can be activated in major tissues from mice rendered septic by CLP (Fig. 1A,B). Sham-operated control mice exhibited undetectable or marginal levels of STAT3 phosphorylation in lung, liver, kidney, and heart tissues. STAT3 phosphorylation profoundly increased in all tissues after CLP in a time-dependent manner, although the time course of increases in STAT3 phosphorylation was somewhat different among tissues. STAT3 activation apparently involves multiple pathways, including JAKs and Src^33,34^. When activation of JAK2 and Src following sepsis was estimated by their phosphorylation in liver tissues, the phosphorylated levels of JAK2, but not of Src, were significantly increased 6–18 h after CLP (Fig. 1C,D).

**Figure 2:**
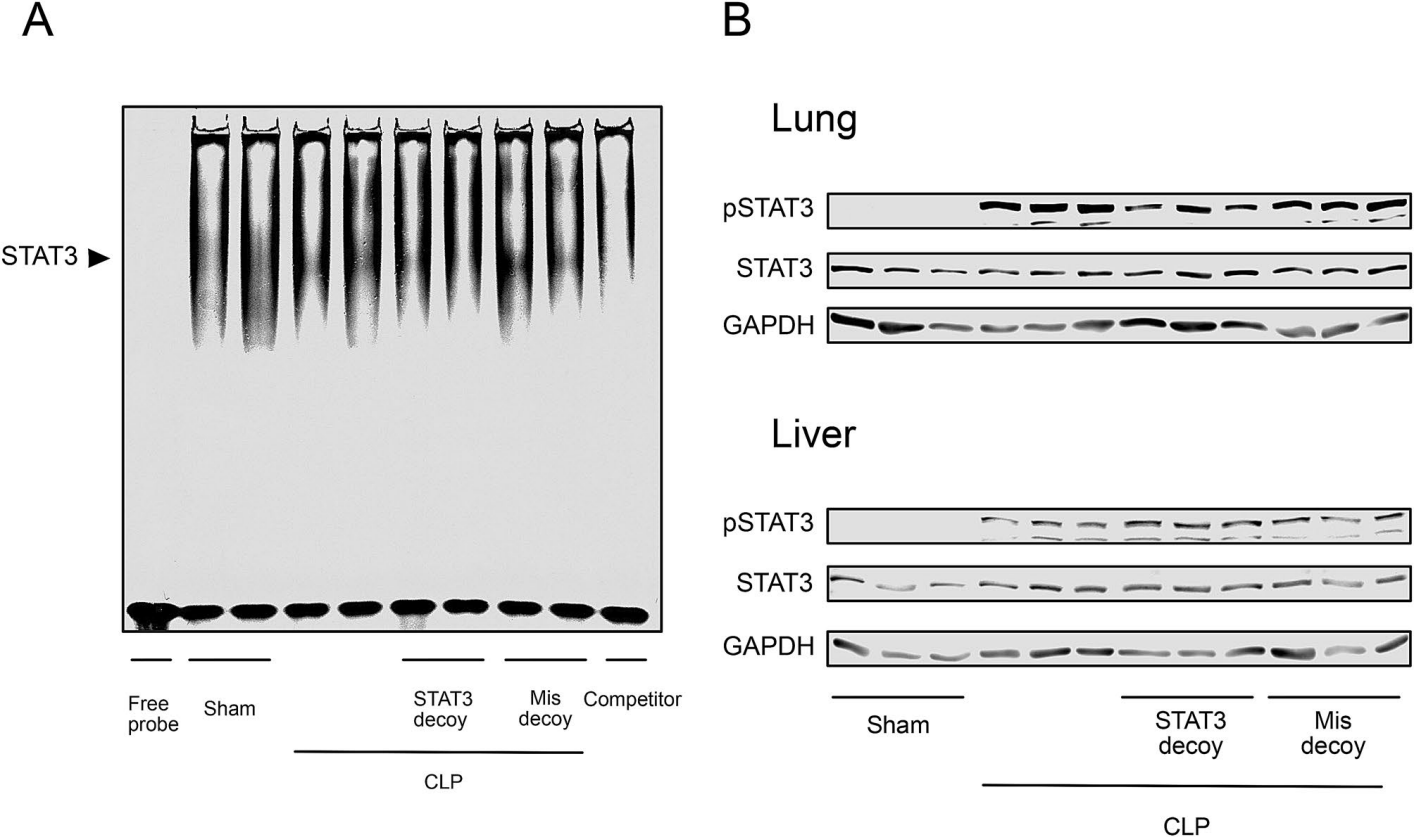
Effect of STAT3 decoy ODN transfection on STAT3 activation in mouse tissues following CLP-induced sepsis. (**A**) Gel mobility shift assay for STAT3 binding activity in lung tissues from sham-operated control and CLP mice at 18 h after surgery. The induced STAT3 shift bands are indicated. Specificity of STAT3 was determined by adding a substantial excess of unlabeled competitor. The inhibitory effect of transfection of STAT3 decoy ODNs, but not of mismatched decoy ODNs, on DNA-binding activity of STAT3 is presented. The binding activity bands of STAT3 were analyzed by densitometry. Shown are representative images from two independent experiments in which the same results were obtained. (**B**) Immunoblot analysis showing lack of effect of STAT3 decoy ODN transfection on phosphorylation of STAT3 in lung and liver tissues following 18-h CLP. GAPDH served as loading control.

The DNA binding of STAT3 in lung tissues was assessed by electrophoretic mobility shift assay (Fig. 2A). The binding activity levels of STAT3 were strikingly increased in mice 18 h after CLP as compared with those in sham-operated mice. The increase in STAT3 binding activity was nearly completely eliminated in lungs from the animals in which double-stranded STAT3 decoy ODNs, but not mismatched decoy ODNs, were introduced by intravenous injection using the transfer system based on atelocollagen, suggesting that our transfer of STAT3 decoy ODNs can efficiently inhibit activity of this transcription factor into tissues. However, transfection of STAT3 decoy ODNs was without effect on phosphorylation levels of STAT3 in lung and liver tissues from CLP-induced septic mice (Fig. 2B).

### STAT3 decoy ODN transfection on sepsis-induced pro-inflammatory cytokine and HMGB1 upregulation

**Figure 3:**
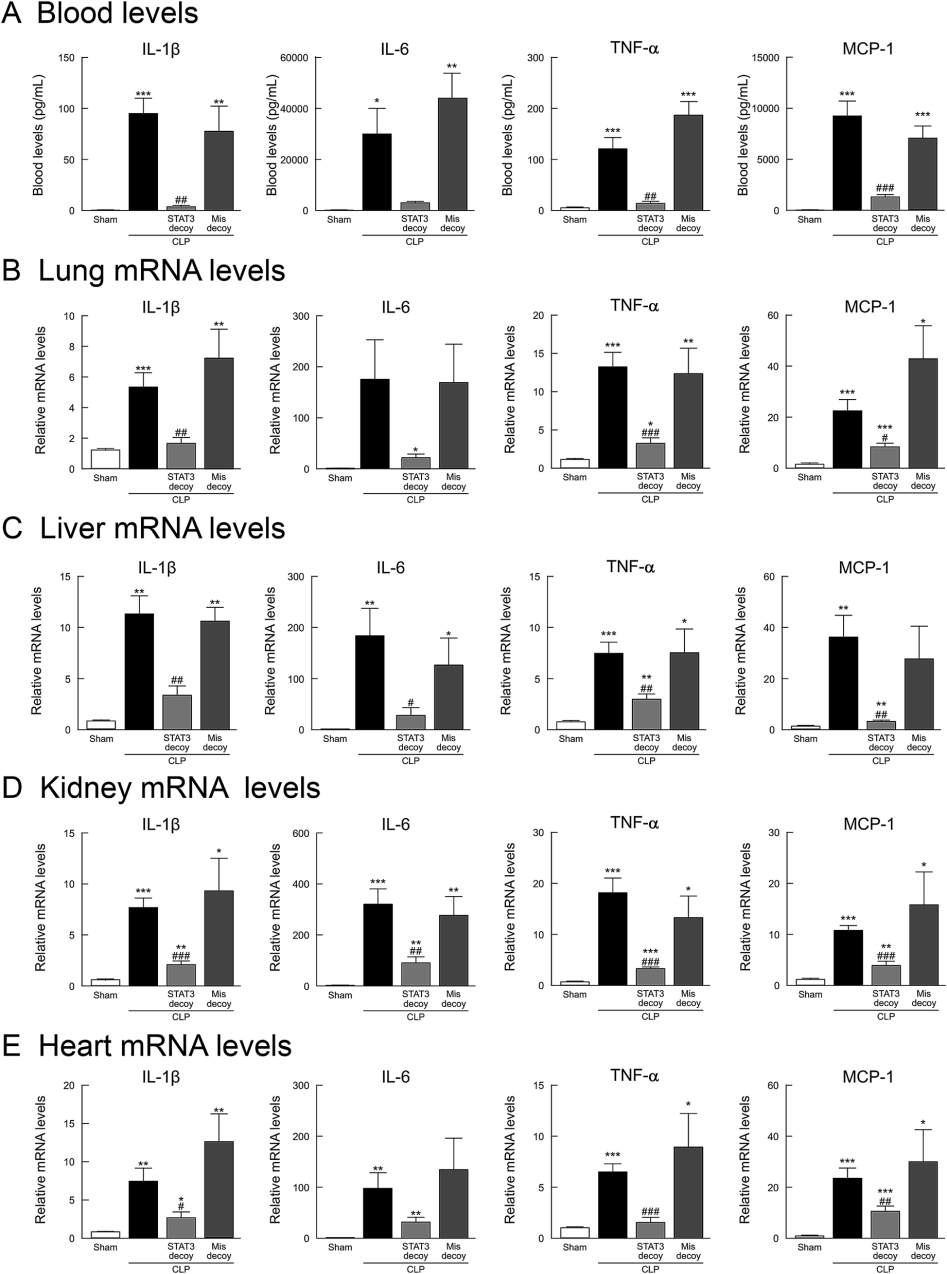
Effect of STAT3 decoy ODN transfection on elevated pro-inflammatory cytokines in mice following CLP-induced sepsis. (**A**) Blood levels of IL-1β, IL-6, TNF-α, and MCP-1. The blood was collected 18 h after surgery (*n* = 6), and those cytokine levels were measured by the use of ELISA. (**B**–**E**) Transcription levels of IL-1β, IL-6, TNF-α, and MCP-1 in lung, liver, kidney, and heart tissues. Tissues were harvested 18 h after surgery (*n* = 6). The mRNA levels were quantified by real-time PCR. The values were expressed as a fold increase above sham-operated control normalized GAPDH. *P < 0.05, **P < 0.01, and ***P < 0.001 compared with the respective control (18 h after sham operation). ^#^*P* < 0.05, ^##^*P* < 0.01, ^###^*P* < 0.001 compared with CLP alone.

We examined changes in blood levels of pro-inflammatory cytokines in mice 18 h after surgery using an enzyme-linked immunosorbent assay (ELISA). Sham-operated control mice displayed extremely low levels of the cytokines examined here (Fig. 3A). Following sepsis induction by CLP, the pro-inflammatory cytokines, IL-1β, IL-6, tumor necrosis factor (TNF)-α, and monocyte chemoattractant protein (MCP)-1, showed a marked increase. The increased blood levels of these cytokines were strikingly inhibited by transfection of STAT3 decoy ODNs, but not of mismatched decoy ODNs. When changes in the levels of mRNA of these pro-inflammatory cytokines in lung, liver, kidney, and heart tissues were measured using real-time PCR, these gene levels were greatly upregulated in CLP-induced septic mice (Fig. 3B–E). Treatment with STAT3 decoy ODNs, but not with mismatched decoy ODNs, evidently inhibited their increased mRNA levels in each tissue.

**Figure 4:**
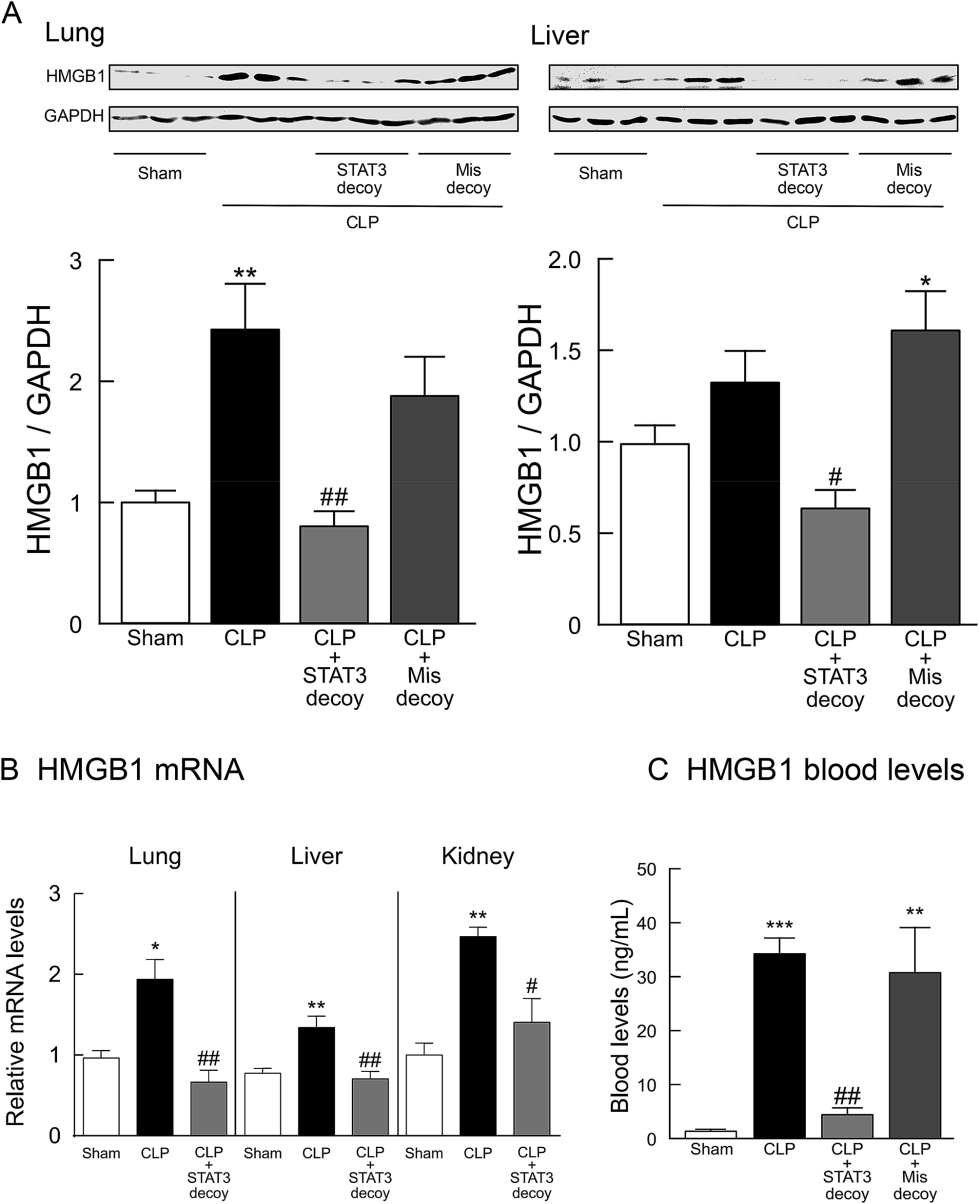
Effect of STAT3 decoy ODN transfection on upregulated HMGB1 levels in mice following CLP-induced sepsis. (**A**) HMGB1 protein levels in lung and liver tissues. Typical Western blots are shown in the top images in each panel. HMGB1 protein levels were normalized to GAPDH (*n* = 6). (**B**) HMGB1 mRNA in lung, liver, and kidney tissues. The mRNA levels of HMGB1 were quantified by real-time PCR and was normalized to GAPDH (*n* = 6). Tissues were harvested 18 h after surgery. (**C**) Serum HMGB1 levels in 18-h CLP mice (*n* = 6). *P < 0.05, **P < 0.01, and ***P < 0.001 compared with the respective control (18 h after sham operation). ^#^*P* < 0.05 and ^##^*P* < 0.01 compared with CLP alone.

High mobility group box 1 (HMGB1) is a nuclear factor that enhances transcriptional activation but also serves as an extracellular cytokine known to be a critical mediator of innate immune responses^35,36^. When HMGB1 protein expression in lung and liver tissues was assessed using Western blot assays, CLP-induced sepsis was found to result in strong immunoreactivity (Fig. 4A). Transfection of STAT3 decoy ODNs resulted in a significant inhibition of the increase in tissue expression of HMGB1 protein (Fig. 4A). The HMGB1 mRNA levels were also upregulated in tissues from mice with CLP-induced sepsis, which was significantly reversed by treatment with STAT3 decoy ODNs (Fig. 4B). We further examined serum HMGB1 levels in CLP-induced septic mice (Fig. 4C). CLP mice displayed a marked elevation in serum HMGB1. Administration of STAT3 decoy ODNs, but not of mismatched decoy ODNs, to CLP mice greatly suppressed the elevated levels of HMGB1.

### STAT3 decoy ODN transfection on organ injury in septic mice

**Figure 5:**
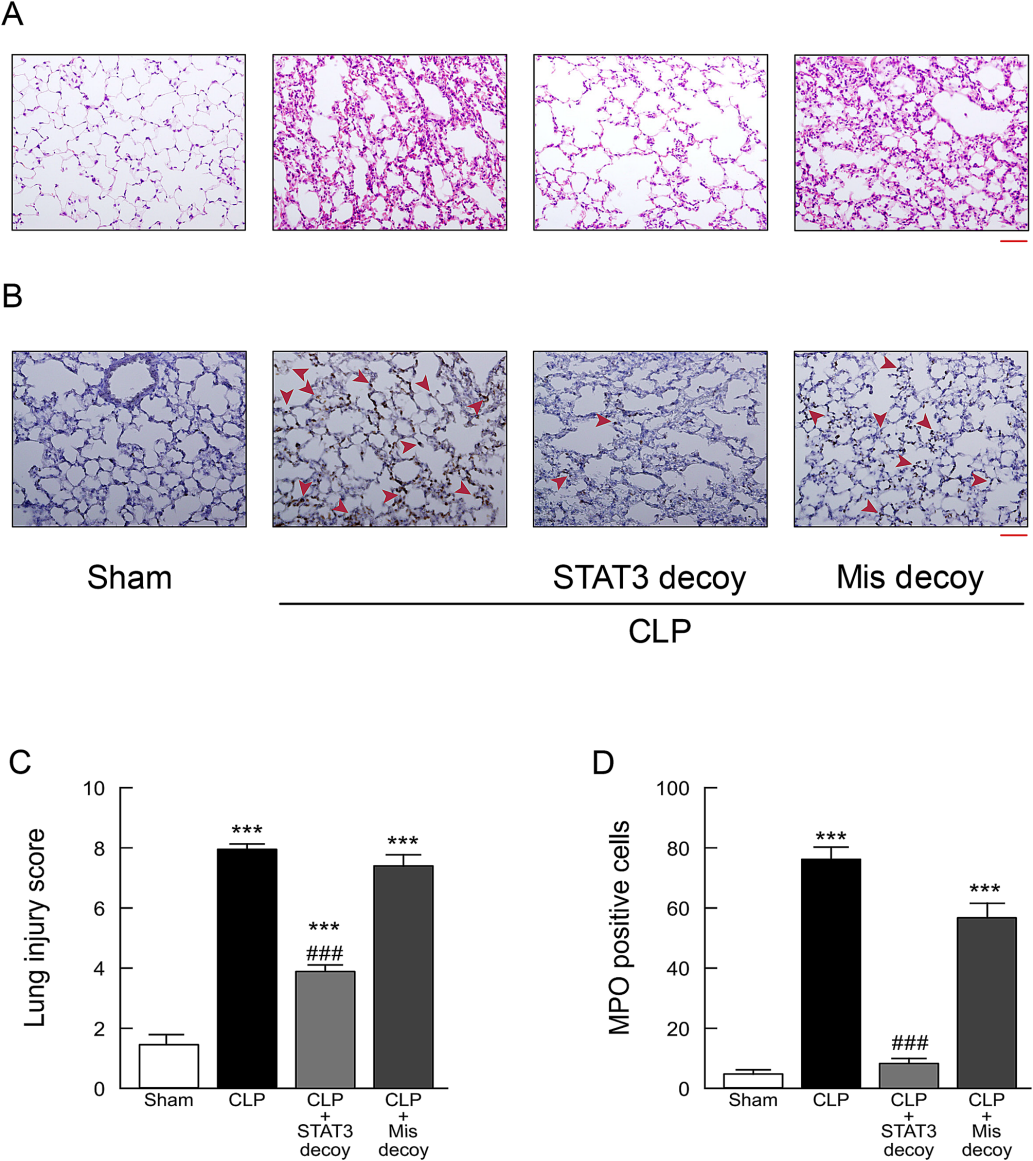
Effect of STAT3 decoy ODN transfection on lung injury in mice following CLP-induced sepsis. Lung tissues were harvested from sham-operated and CLP-induced septic mice 18 h after surgery. (**A**) Lung tissue sections stained with hematoxylin and eosin. (**B**) Lung tissue sections were stained with antibody against MPO followed by peroxidase staining. MPO-positive cells were stained brown and indicated by red arrowheads. Scale bar, 100 μm. (**C**) Semiquantitative analysis of lung tissues by lung injury, which was assessed by scoring from 0 to 4 as described in Methods (*n* = 6). (**D**) Quantitation of MPO-positive cell counts in lung tissues. The average of MPO-positive cell number in five fields per sample was calculated (*n* = 6). ***P < 0.001 compared with the respective control (18 h after sham operation). ^###^*P* < 0.01 compared with CLP alone.

Histopathological examination of hematoxylin and eosin-stained sections of lung tissues revealed that massive infiltration of inflammatory cells, marked congestion of pulmonary edema, disorganized architecture with irregular alveoli, and erythrocytes originating from ruptured capillary vessels in mice 18 h after sepsis induction by CLP (Fig. 5A). Such histological damage in lungs in septic mice was prevented by transfection of STAT3 decoy ODNs. Semiquantitative assessment using lung injury score showed that the score was significantly lowered by administration of STAT3 decoy ODN, but not of mismatched decoy ODNs (Fig. 5C). The animals 18 h after CLP-induced sepsis exhibited markedly increased staining for myeloperoxidase (MPO), an index of neutrophil infiltration, in lung sections (Fig. 5B,D). Treatment with STAT3 decoy ODNs, but not with mismatched decoy ODNs, significantly inhibited the increase in MPO staining in lung tissues.

**Figure 6:**
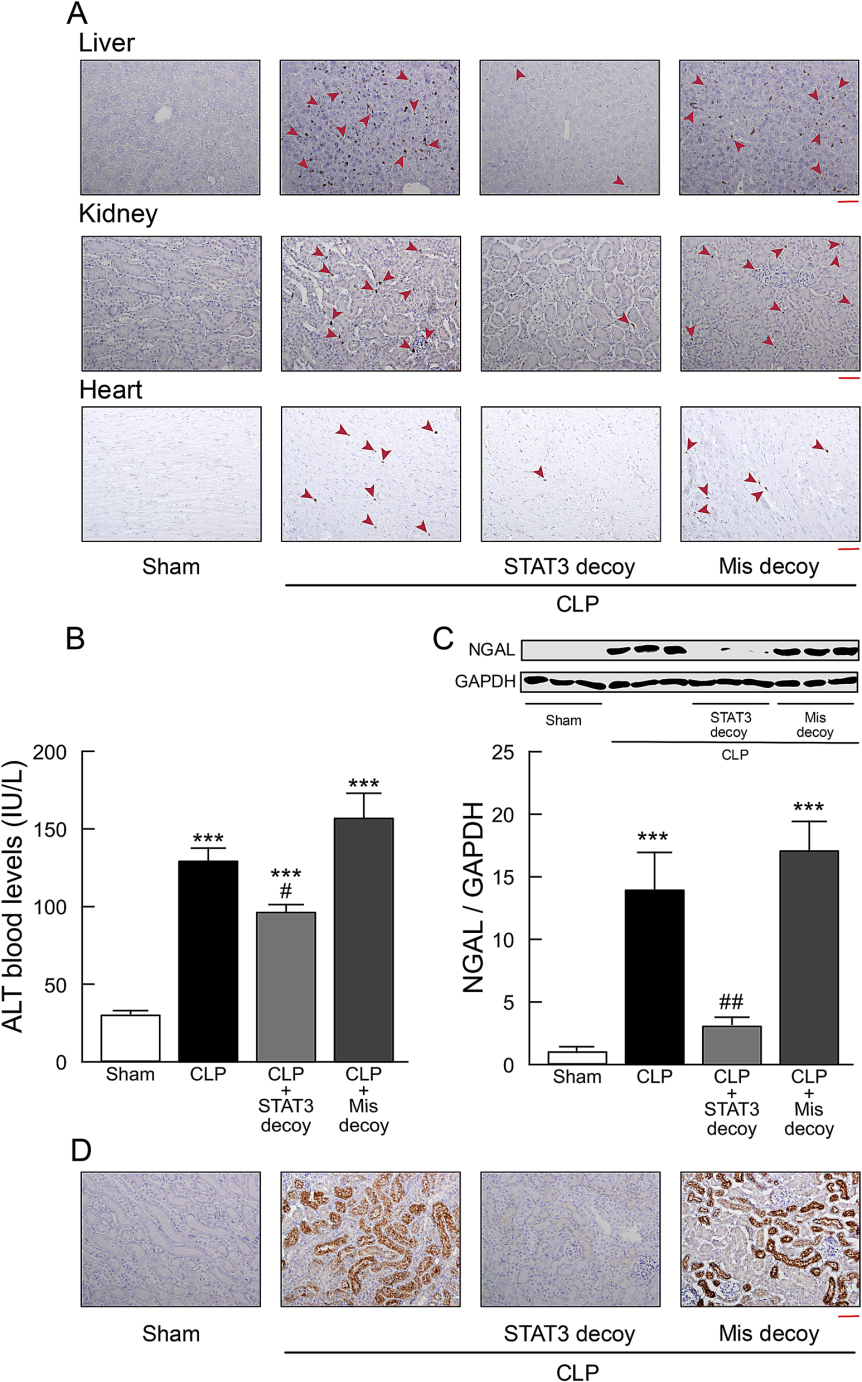
Effect of STAT3 decoy ODN transfection on liver, kidney, and heart injury in mice following CLP-induced sepsis. Tissues and blood samples were taken from sham-operated and CLP-induced septic mice 18 h after surgery. (**A**) Representative micrographs of liver, kidney, and heart sections stained with antibody against MPO followed by peroxidase staining. MPO-positive cells were stained brown and indicated by red arrowheads. Scale bar, 100 μm. The same results were obtained with two other experiments. (**B**) Serum levels of ALT (n = 5). (**C**) Western blots of NGAL protein expression. Typical blots are shown in the top traces. NGAL protein levels were normalized to GAPDH (n = 6). ***P < 0.001 compared with the respective control (18 h after sham operation). #P < 0.05 and ##P < 0.01 compared with CLP alone. (**D**) Kidney tissue sections were stained with anti-NGAL antibody followed by peroxidase staining. Scale bar, 100 μm. Shown are representative images from three independent experiments.

Significantly increased MPO staining was also observed in liver, kidney, and heart sections from CLP mice, which was prevented by transfection of STAT3 decoy ODNs, but not of mismatched decoy ODNs (Fig. 6A).

A marked elevation in serum levels of alanine aminotransferase (ALT), a functional indicator for liver damage, was observed in mice following induction of sepsis by CLP (Fig. 6B). The elevation in these serum aminotransferase levels after sepsis was significantly decreased in CLP mice given STAT3 decoy ODN.

Detection of neutrophil gelatinase-associated lipocalin (NGAL) was used as a biomarker of acute kidney injury after CLP-induced sepsis. Western blot analysis showed that the increase in NGAL in kidney tissues of septic mice was significantly reduced by transfection of STAT3 decoy ODNs, but not of mismatched decoy ODNs (Fig. 6C). In kidney sections from CLP mice, NGAL staining intensity was strongly increased (Fig. 6D). In sections from CLP mice given STAT3 decoy ODNs, NGAL staining was markedly diminished.

### STAT3 decoy ODN transfection on animal survival after CLP

**Figure 7:**
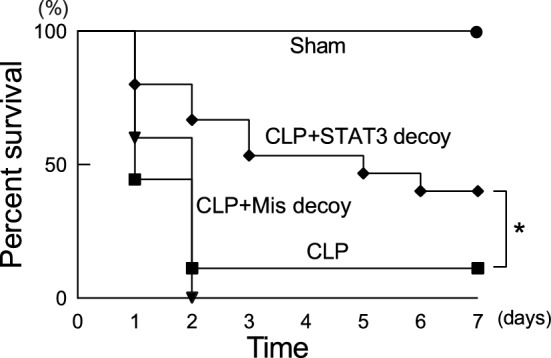
Effect of STAT3 decoy ODN transfection on mortality in mice after CLP. Mice were subjected to sham operation or CLP. STAT3 decoy ODNs or mismatched decoy ODNs were injected intravenously at 1 h after CLP. Survival was recorded for 7 days. Fifteen to 18 animals were used for each group. *P < 0.05 (log-rank test).

Whether STAT3 decoy administration can improve survival of mice with CLP-induced sepsis was addressed (Fig. 7). No deaths occurred in sham-operated control animals. More than 90% of the animals subjected to CLP died throughout the 7 days of observation. A significant survival benefit was observed in the animals given STAT3 decoy ODN at 1 h after CLP. Mismatched decoy ODNs were without effect on animal survival after CLP.

### Effect of stattic on pro-inflammatory cytokine upregulation and organ injury in septic mice

We finally investigated whether the STAT3 inhibitor stattic could mimic the beneficial effects of STAT3 decoy ODN transfection on pro-inflammatory cytokine upregulation and organ injury in mice with CLP-induced sepsis. Stattic was intraperitoneally injected 1 and 12 h after CLP surgery at a dose of 25 mg/kg^37^.

**Figure 8:**
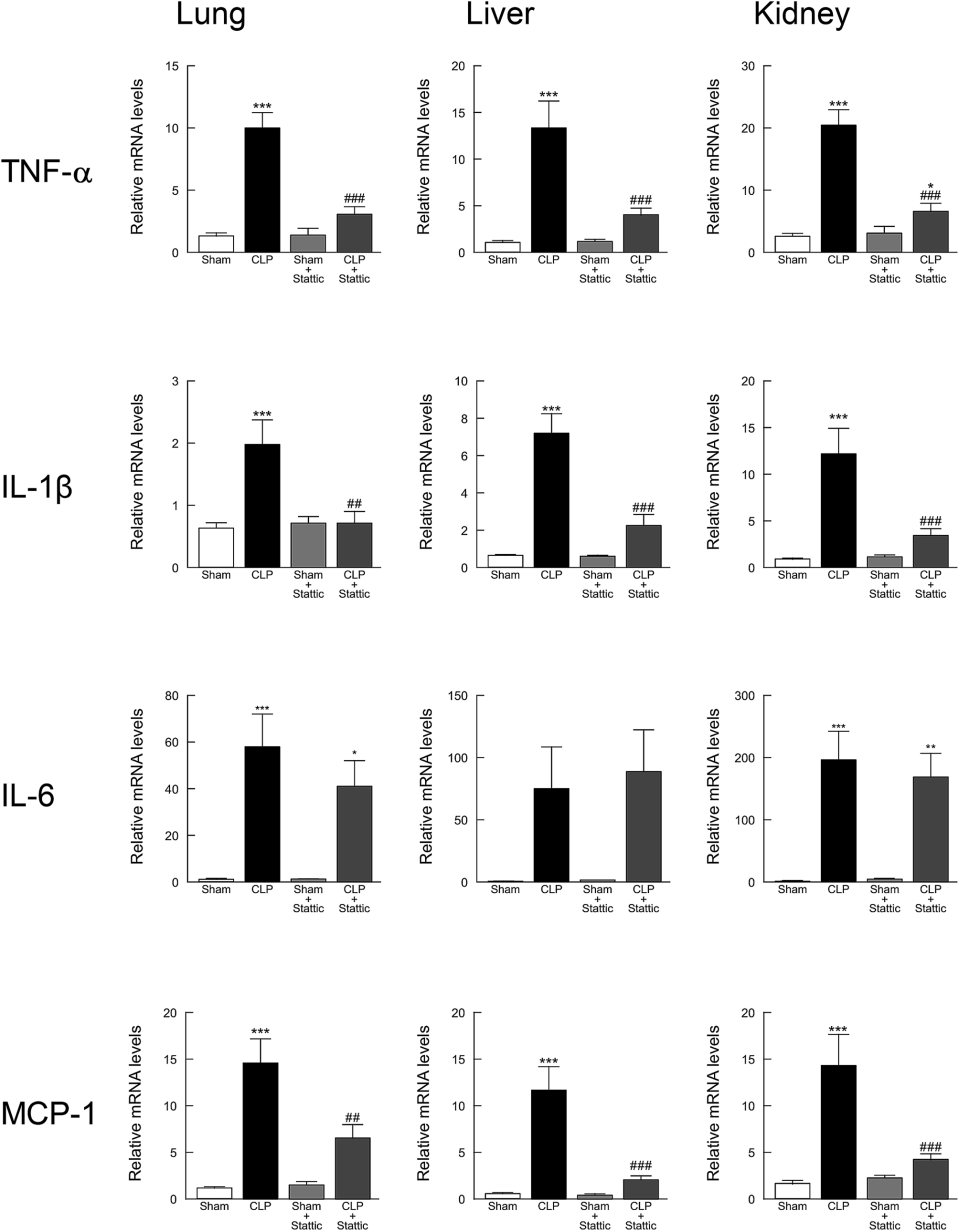
Effect of stattic treatment on elevated pro-inflammatory cytokines in mice following CLP-induced sepsis. Lung, liver, and kidney tissues were harvested 18 h after surgery and the mRNA levels of IL-1β, IL-6, TNF-α, and MCP-1 were quantified by real-time PCR (*n* = 9). The values were expressed as a fold increase above sham-operated control normalized GAPDH. *P < 0.05, **P < 0.01, and ***P < 0.001 compared with the respective control (18 h after sham operation). ^##^*P* < 0.01 and ^###^*P* < 0.001 compared with CLP alone.

When changes in mRNAs of pro-inflammatory cytokines in lung, liver, and kidney tissues were investigated using real-time PCR, the greatly increased mRNA expression levels of TNF-α, IL-1β, and MCP-1 at 18 h after CLP-induced sepsis were significantly suppressed by stattic administration (Fig. 8). However, administration of stattic did not substantially affect the elevated level of IL-6 mRNA in CLP mouse tissues.

**Figure 9:**
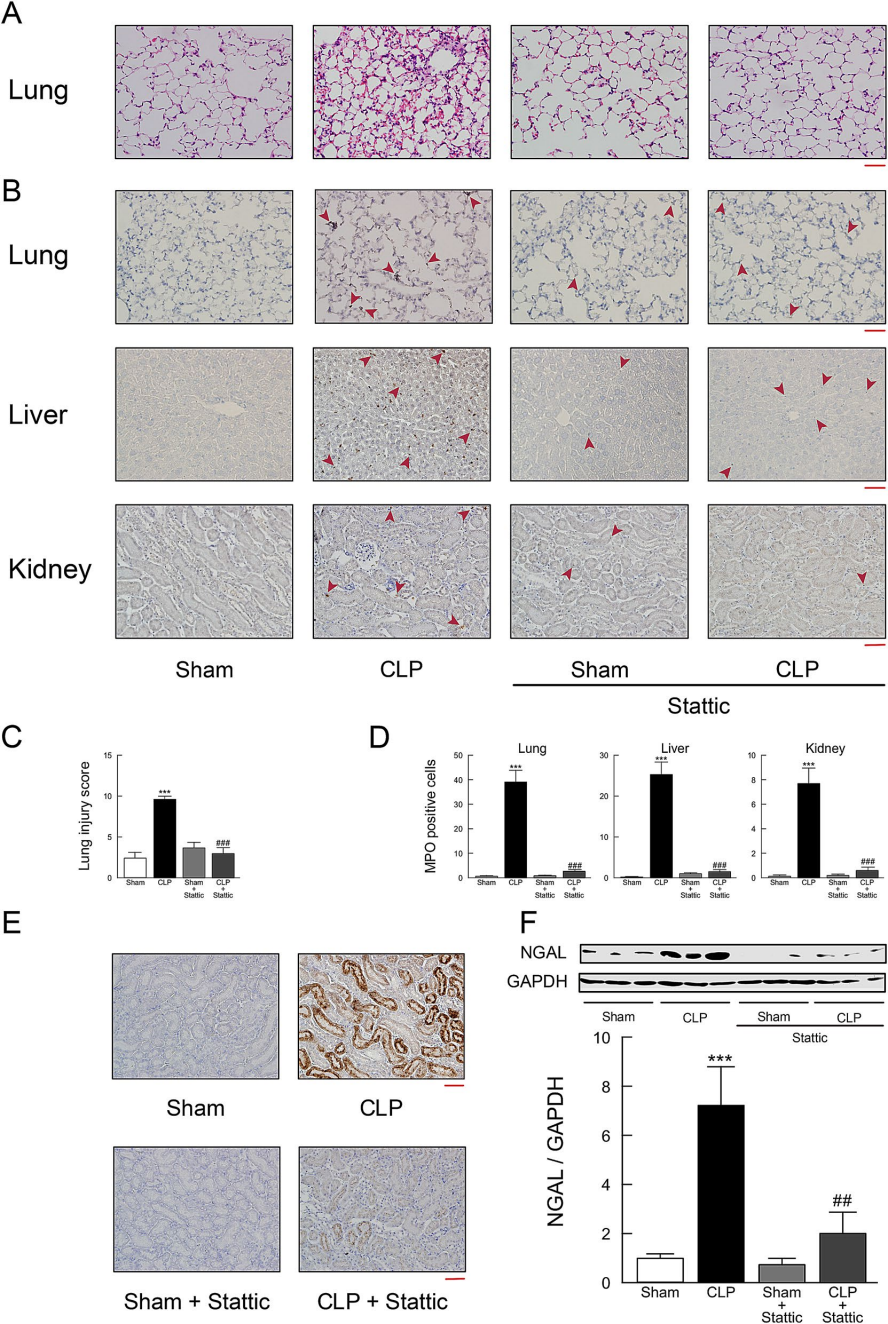
Effect of stattic treatment on tissue injury in mouse tissues following CLP-induced sepsis. Lung, liver, and kidney tissues were harvested 18 h after surgery. (**A**) Lung tissue sections stained with hematoxylin and eosin. (**B**) Lung, liver, and kidney tissue sections stained with antibody against MPO followed by peroxidase staining. MPO-positive cells were stained brown and indicated by red arrowheads. Scale bar, 100 μm. (**C**) Semiquantitative analysis of lung tissues by lung injury, which was assessed by scoring from 0 to 4 as described in Methods (*n* = 6). (**D**) Quantitation of MPO-positive cell counts in lung, liver, and kidney tissues. The average of MPO-positive cell number in five fields per sample was calculated (*n* = 6). (**E**) Kidney tissue sections stained with anti-NGAL antibody followed by peroxidase staining. Scale bar, 100 μm. Shown are representative images from three independent experiments. (**F**) Western blots of NGAL protein expression. Typical blots are shown in the top traces. NGAL protein levels were normalized to GAPDH (*n* = 6). ***P < 0.001 compared with the respective control (18 h after sham operation). ^##^*P* < 0.01 and ^###^*P* < 0.01 compared with CLP alone.

Treatment with stattic resulted in significant protections from histological damage of lungs (Fig. 9A,C), increased neutrophil influx in lung, liver, and kidney tissues (Fig. 9B,D), and increased NGAL production in the kidney (Fig. 9E,F) in CLP mice.

### Discussion

In this study, we demonstrate that STAT3, one of the STAT family members that has been implicated to be a key transcription factor in both immunity and inflammatory pathways^23,24^, is greatly activated in major end-organ tissues of mice with CLP-induced sepsis. Our observation is consistent with and extends other previous reports showing that STAT3 is rapidly activated in lungs after LPS administration in mice^30,38^. When polymicrobial sepsis was induced by CLP in mice, STAT3 activation occurred at 3 h, with maximal activation at 6–12 h, although the kinetics of STAT3 activation after CLP appeared to be somewhat different among tissues: STAT3 phosphorylation was observed to be long-lasting in lung tissues but to show a temporary rise in liver, heart, and kidney tissues. STAT3 activation is largely mediated by the JAK kinase family, and the Src family kinases may be also involved^20,21^. We found that JAK2, but not Src, was significantly activated in mouse tissues after CLP. This suggests that JAK2 may serve as an upstream kinase of STAT3 during CLP-induced sepsis.

We generated phosphorothioate double-stranded ODNs, which contains two binding sites for STAT3 recognized in the stem region in the DNA structure, and in vivo introduced into mice with CLP-induced sepsis. To deliver those ODNs into organ tissues, we used the transfer system based on atelocollagen, a biomaterial with a porous structure. This method has been confirmed to show efficient delivery of small interfering RNAs and oligonucleotides^39,40^. Our in vivo transfection of STAT3 decoy ODNs greatly inhibited the increase in STAT3 binding activity in lungs of CLP-induced septic mice, as indicated by an electrophoretic mobility shift assay, representing the successful in vivo transfer of a sufficient quantity of STAT3 decoy ODNs into tissues under sepsis conditions. In line with the view that STAT3 decoy ODN operates to trap activated STAT3, which dimerizes and translocates into the nucleus, in the cytoplasm, its transfection was found to be without effect on STAT3 phosphorylation associated with CLP-induced sepsis.

We showed that in vivo transfection of STAT3 decoy ODNs minimized the development of major end-organ injury in mice with CLP-induced sepsis and resulted in a prominent survival advantage in the animals after CLP. The impact of ODN decoy to STAT3 on mortality in this rodent sepsis model appears to be more pronounced when compared with those of other transcription factors, such as NF-κB and AP-1, which are major participants in modulating the transcription of the immunoregulatory mediators. We have previously shown that transfection of NF-κB decoy ODNs has a significant but small effect on the survival, suggesting that suppression of NF-κB may be of limited importance in preventing septic mortality^16,17^. In this regard, it is noteworthy that NF-κB not only plays a role as a survival factor, responsible in part for “turning on” genes that could block cell death by apoptosis, but also works essentially in maintaining normal host defense mechanisms^41,42^. Administration of ODN decoy to another transcription factor AP-1 has led to a significant long-term survival in mice after CLP at the end of observation period possibly resulting from death from apoptosis-associated immunoparalysis and tissue parenchymal damage, but has been not substantially improve a sharp decline in survival in the course of 36 h after surgery^18^.

The benefit of STAT3 decoy ODN transfection to prevent the development of major end-organ injury, leading to a significant survival advantage, may be partly attributed to the downregulation of pro-inflammatory cytokine and chemokine overproduction in CLP mice. Our findings imply that STAT3 is critical for the production of different pro-inflammatory mediators, including TNF-α, IL-1β, IL-6, and MCP-1, in CLP-induced sepsis, although STAT3 would work coordinately with other transcriptional co-activators or transcription factors. The downregulating effect on overproduction of pro-inflammatory cytokines and chemokines has been also found when NF-κB or AP-1 decoy ODNs are applied to CLP mice despite their survival advantages being different from that of STAT3 decoy ODNs as mentioned above. Furthermore, it is best to fully aware that eritoran, which antagonizes LPS signaling and thereby is expected to inhibit the extreme reaction associated with excessive and uncontrolled production of pro-inflammatory cytokines and chemokines, fails to show a significant benefit in phase III clinical trials of septic patients with organ dysfunction^43^.

In the present study, we revealed that STAT3 plays a pivotal role in transcriptional control of HMGB1. HMGB1 is constitutively expressed in quiescent macrophage/monocytes, stored in the nucleus, and actively secreted from stimulated immune cells^36^. HMGB1 mRNA levels were greatly upregulated in major organ tissues of CLP mice, and the upregulation of HMGB1 transcript was strongly suppressed by STAT3 decoy ODN transfection. Furthermore, transfection of STAT3 decoy ODNs significantly inhibited the increase in tissue levels of HMGB1 protein seen in CLP mice. The parallel behavior of mRNA and protein expression levels of HMGB1 when ODN decoy was applied implies that HMGB1 is regulated in a transcription manner by STAT3. HMGB1 is a nuclear factor that enhances transcriptional activation but also serves as an extracellular cytokine known to be a critical mediator of innate immune responses^35,36^. Outside the cells, HMGB1 can activate pro-inflammatory cytokine release from immune cells, including TNF-α and IL-1β^44^. Thus, the ability of STAT3 decoy ODN to regulate HMGB1 may contribute to its suppressive effect on overproduction of pro-inflammatory and chemotactic cytokines. HMGB1 is considered as a critical regulator of sepsis severity. We found that administration of STAT3 decoy ODNs greatly suppressed the excessive rise in serum HMGB1 levels in CLP-induced septic mice. Blood HMGB1 concentrations are elevated in patients with sepsis^45^. Septic patients who succumbed to infection had higher serum HMGB1 levels than those who survived^46^. Our results thus indicate that HMGB1 may be one of the potential target molecules for the therapeutic strategy using STAT3 decoy ODNs in sepsis treatment. However, it would be important to promote a greater understanding of the role of HMGB1 in the pathological process of the development of sepsis organ injury.

We demonstrated that pharmacological intervention with the STAT3 inhibitor stattic mimicked the beneficial effect of STAT3 decoy ODNs on major end-organ tissue injury in mice with CLP-induced sepsis. Unlike STAT3 decoy ODN transfection, however, treatment with stattic failed to reduce the upregulation of IL-6 in CLP mice, although the ability of stattic to inhibit the increases in TNF-α, IL-1β, and MCP-1 was similar to that of STAT3 decoy ODNs. Stattic predominantly acts as a STAT3 tyrosine phosphorylation inhibitor rather than a STAT3 pathway inhibitor. Thus, this compound can bind to the SH2 domain of STAT3 and prevent Tyr-705 phosphorylation^32^. While numerous innate immune cytokines can activate STAT3, STAT3 is preferentially activated by IL-6 via the JAK signaling pathway^47^. As such, given the IL-6/JAK2/STAT3 signaling axis, the persisting upregulation of IL-6 in the presence of stattic may result from a negative feedback loop which is initiated by the breakdown in the step that STAT3 becomes activated through tyrosine phosphorylation. Alternatively, the use of STAT3 inhibitors, such as stattic, in sepsis treatment might unmask IL-6-mediated but STAT3-independent unexpected harmful reactions.

In conclusion, our present work provides evidence that STAT3 plays a critical role in the pathophysiology of sepsis. We also demonstrate that transfection of STAT3 decoy ODNs affords protection against the development of sepsis-driven major end-organ injury and leads to a prominent survival advantage in mice after CLP. While appreciating that several crucial issues, including safety and adverse effects, have to be addressed in further study, our results suggest that STAT3 suppression with the use of transcription decoy strategy may represent a novel and efficacious therapeutic option for sepsis treatment.

## Methods

### Animal models and the studies of sepsis

All animal studies and experiments were approved by the Research Facility of Toyama University (A2018MED-30) and the Animal Research Facility of Nagoya University (20327, 31345). All animal studies and the methods were carried out in accordance with the relevant guidelines and regulations in the manuscript.

The surgical procedure to generate CLP-induced sepsis was conducted as described in our previous reports^48–50^. In brief, male BALB/c mice (Sankyo Lab Service, Tokyo Japan), 8–12 weeks old, were anesthetized with 3–4% sevoflurane by inhalation, and a middle abdominal incision was made. The cecum was mobilized, tightly ligated (1 cm from the cecum tip), punctured twice with a 21-gauge needle, and gently squeezed to expel small amounts of feces. Then, the bowel was repositioned to the peritoneal cavity, and the laparotomy site was closed with sterile suture (the skin and muscle were sutured separately). Sham-operated control underwent the same procedure except for ligation and puncture of the cecum. Both groups of animals were fed the same diet and water ad libitum. The animals after CLP surgery were all lethargic, showed lack of interest in their environment, displayed piloerection, and had crusty exudates around their eyes, as contrasted with sham-operated animals that were healthy, moving freely and eating^50^. Normal saline (0.5 ml) was given subcutaneously to all mice immediately after surgery. Unless stated otherwise, the animals at 18 h after surgery were used for the experiments, in which blood samples were collected and lung, heart, liver, and kidney tissues were harvested from mice treated with ketamine (80–100 mg/kg) and xylazine hydrochloride (10 mg/kg). All experimental data were analyzed in a blinded fashion.

### Preparation and delivery of ODNs

The following sequences of phosphorothioate double-stranded ODN against STAT3 binding site and of mismatched ODN were used in this study, which were the same as described previously^51^: 5′-CATTTCCCGTAAATC-3′ and 3′-GTAAAGGGCATTTAG-5′ for STAT3 decoy ODN (consensus sequences are underlined); 5′-CATGTTGCCTATATC-3′ and 3′-GTACAACGGATATAG-5′ for mismatched decoy ODN. It is documented that STAT3 decoy ODNs are cell-permeable and a potent inhibitor of STAT3 with half-lives > 12 h and may reach to 12 days^52^. For in vivo transfer of ODNs, AteloGene Systemic Use kit (KOKEN, Tokyo, Japan) was employed. AteloGene consists of a system mediated by atelocollagen, a solubilized collagen obtained by protease treatment. Atelocollagen and oligonucleotides form a complex of nanosized particles and the complex allows oligonucleotides to be delivered efficiently into organs and tissues via intravenous administration^40^. The atelocollagen drug delivery technology protects the ODNs from degradation by host nucleases and ensures a sustained lifetime effect in living body^40^. Sterile distilled water (200 μl) containing synthetic ODNs (8 nmol/animal) was infused into the tail vein of an anesthetized mouse over 20 s at room temperature at 60 min after surgery.

### Western blot analysis

After being removed and rinsed in sterilized phosphate buffered saline on ice, tissues were homogenized and then centrifuged at 18,000×*g* for 10 min at 4 °C, and the resulting supernatants were collected. The proteins in the supernatant were measured using BCA Protein Assay Kit (Nacalai Tesque, Kyoto, Japan). Immunoblotting was performed as described previously^48,50,53^. Samples (30–50 μg of protein) were electrophoresed on 10–14% SDS-PAGE and transferred to PVDF membrane. The membrane was blocked for 1 h at room temperature in Odyssey blocking buffer (LI-COR Bioscience, Lincoln, NE, USA) followed by overnight incubation with primary antibody at 4 °C. Primary antibody detection was performed with IRDye-labeled secondary antibodies. Fluorescent of IR-Dye was analyzed by Odyssey CLx Infrared Imaging System (LI-COR Bioscience).

The following antibodies, which are commercially available, were used: anti-human STAT3 mouse monoclonal antibody (1:1,000; Cell Signaling, Danvers, MA), anti-mouse phospho-STAT3 (Thy-705) rabbit monoclonal antibody (1:1,000; Cell Signaling), anti-human JAK2 mouse monoclonal antibody (1:300; Santa Cruz Biotechnology, Santa Cruz, CA, USA), anti-human phospho-JAK2 (Tyr-1007/Tyr-1008) rabbit polyclonal antibody (1:500; Cell Signaling), anti-human Src rabbit monoclonal antibody (1:1,000; Cell Signaling), anti-human phospho-Src (Tyr-416) rabbit monoclonal antibody (1:1,000; Cell Signaling), anti-human HMGB1 rabbit monoclonal antibody (1:1,000; Cell Signaling), anti-mouse NGAL rabbit polyclonal antibody (1:1,000; Abcam, Cambridge, UK), and anti-human glyceraldehyde-3-phosphate dehydrogenase (GAPDH) chicken polyclonal antibody (1:3,000; EMD Millipore, Billerica, MA).

### Electrophoretic mobility shift assay

Nuclear protein extracts from freshly isolated lungs were obtained with a commercially available nuclear extraction kit (Sigma-Aldrich, St. Louis, MO, USA) as described in the manufacturer's manual. Electrophoretic mobility shift assays were carried out with Odyssey Infrared electrophoretic mobility shift assay kit (LI-COR, Lincoln, NE) according to the manufacturer's instructions. Double-stranded IRDye 700 infrared dye-labeled oligonucleotides with consensus sequences of STAT3 (5′- CATTTCCCGTAATC-3′ and 3′-GTAAAGGGCATTTAG-5′) (Integrated DNA Technologies, Coralville, IA, USA) were used for visualization.

### RNA extraction and quantitative real-time PCR

Total RNA was isolated from tissues with the use of Sepazol-RNA I Super G (Nacalai Tesque) according to the manufacturer’s manual. ReverTra Ace qPCR RT Master Mix (Toyobo, Osaka, Japan) was used for the reverse transcription reaction, and real-time PCR analyses were performed using PowerUp SYBR Green Master Mix (Thermo Fisher Scientific, Rockford, IL, USA), as described in the manufacturers’ instructions. Values were normalized to the housekeeping gene GAPDH according to the manufacturer’s protocol (MX3000P real-time PCR system; Agilent Technologies Inc., Santa Clara, CA, USA). Additional details are described by our laboratory^37,49,50^. The PCR primers were designed as follows: forward 5′- GTTCTATGGCCCAGACCCTCAC-3′ and reverse 5′-GGCACCACTAGTTGGTTGTCTTTG-3′ for TNF-α, forward 5′-TCCAGGATGAGGACATGAGCAC-3′ and reverse 5′-GAACGTCACACACCAGCAGGTTA-3′ for IL**-**1β, forward 5′-CCACTTCACAAGTCGGAGGCTTA-3′ and reverse 5′-GCAAGTGCATCATCGTTGTTCATAC-3′ for IL-6, forward 5′- CTCCAGCCTACTCATTGGGATCA-3′ and reverse 5′- GCATCCACGTGTTGGCTCA-3′ for MCP-1, forward 5′-AGCCCTGTCCTGGTGGTATTTTCAA-3′ and reverse 5′-GCTGTGCACCAACAAGAACCTGC-3′ for HGMB1, and forward 5′-TGTGTCCGTCGTGGATCTGA-3′ and reverse 5′-TTGCTGTTGAAGTCGCAGGAG-3′ for GAPDH.

### Blood analysis

Cardiac puncture allowed the collection of appropriate amounts of blood from a single animal. Blood levels of TNF-α, IL-1β, IL-6, and MCP-1 were measured by the use of commercially available ELISA kit (R&D Systems, Minneapolis, MN) according to the manufacture’s instructions. To measure the concentrations of HMGB1 in serum samples, HMGB1 Detection Kit (Chondrex, Redmond, WA) was used. The quantitative determination of ALT in serum was made on Hitachi 7,180 Biochemistry Automatic Analyzer (Hitachi High-Technologies, Tokyo, Japan). The plate was read on a microplate reader (Nippon-InterMed, Tokyo, Japan). Assays were performed in duplicate.

### Histological examination and immunohistochemistry

Tissues were fixed by immersion in 10% buffered formaldehyde overnight, embedded in paraffin, and cut into 4-μm-thick sections. After deparaffinization, slides were stained with hematoxylin and eosin by standard methods. All the histological studies were performed in a blinded fashion. A semiquantitative morphometric analysis of lung injury was performed by scoring from 0 to 4 (none, light, moderate, severe, very severe) for the following categories: neutrophil infiltration, pulmonary edema, and disorganization of lung parenchyma and hemorrhage^18,54^. A total lung injury score was calculated by adding the individual scores in every animal and averaging the total values in each group.

Immunohistological examination was performed as described previously^50,53^. Tissue Sections (4 μm) were rehydrated, and endogenous peroxidases were quenched with 3% hydrogen peroxide. Slides were then incubated overnight at 4 °C with primary antibodies for MPO (1:200 dilution; Abcam) or NGAL (1:2000 dilution; Abcam). All sections were incubated with Histone Simple Stain Mouse MAX PO(R) (Nichirei Biosciences, Tokyo, Japan) including the secondary antibody which is reduced to Fab fragment. Sections were developed with 3,3′-diaminobenzidine and counterstained with hematoxylin.

### Survival studies in sepsis

Additional groups of mice underwent sham or CLP surgery and were included in survival studies. The animals subjected to CLP were randomly divided into three groups. At 60 min after CLP, one group of the animals was given only saline injections. The other groups received intravenous administration of STAT3 or its mismatched decoy ODNs. The animals were allowed free access to food and water. Postoperative survival was assessed every 6 h for the first 48 h. The survival time of each animal was recorded for 7 days.

### Statistical analysis

Numerical data are expressed as means ± SEM. Data were analyzed using Prism software (ver.6; GraphPad Software Inc., San Diego, CA, USA). Statistical assessment of the data was made by Student’s unpaired *t*-test or one-way ANOVA followed by Tukey’s multiple comparison test. Survival curves were estimated for each group, considered separately, using the Kaplan–Meier method and compared statistically using the log-rank test. Differences at *P* < 0.05 were considered statistically significant.

## Supplementary information

Supplementary information.
