# Supplementary material for: Beneficial effect of STAT3 decoy oligodeoxynucleotide transfection on organ injury and mortality in mice with cecal ligation and puncture-induced sepsis
Source: Sci Rep. 2020 Sep 17;10:15316. doi: 10.1038/s41598-020-72136-x (PMC7498613; doi:10.1038/s41598-020-72136-x)
Supplement: Supplementary file 1 — Supplementary information. [file 41598_2020_72136_MOESM1_ESM.pdf]

Supplementary information

Beneficial effect of STAT3 decoy oligodeoxynucleotide transfection on organ injury and mortality in mice with cecal ligation and puncture-induced sepsis

Samar Imbaby[1], Naoyuki Matsuda[2], Kengo Tomita[1], Kohshi Hattori[3], Sailesh Palikhe[1], Hiroki Yokoo[4], & Yuichi Hattori[1,5]

[1]Department of Molecular and Medical Pharmacology, Graduate School of Medicine and Pharmaceutical Sciences, University of Toyama, Toyama, Japan

[2]Department of Emergency and Critical Care Medicine, Nagoya University Graduate School of Medicine, Nagoya, Japan

[3]Department of Anesthesiology and Pain Relief Center, University of Tokyo, Tokyo, Japan

[4]Department of Health and Nutritional Sciences, Faculty of Health Promotional Sciences, Tokoha Gakuen University, Hamamatsu, Japan

[5]The Research Institute of Cancer Prevention, Health Sciences University of Hokkaido, Tobetsu, Japan

Correspondence: Naoyuki Matsuda. Department of Emergency and Critical Care Medicine, Nagoya University Graduate School of Medicine, Nagoya 466-0065, Japan.

TEL: +81-52-744-2995, FAX: +81-52-744-2978

E-mail: nmatsuda@med.nagoya-u.ac.jp

Current address for Kengo Tomita: Institute of Technology, Shimizu Corporation, Tokyo, Japan

Fig. S1

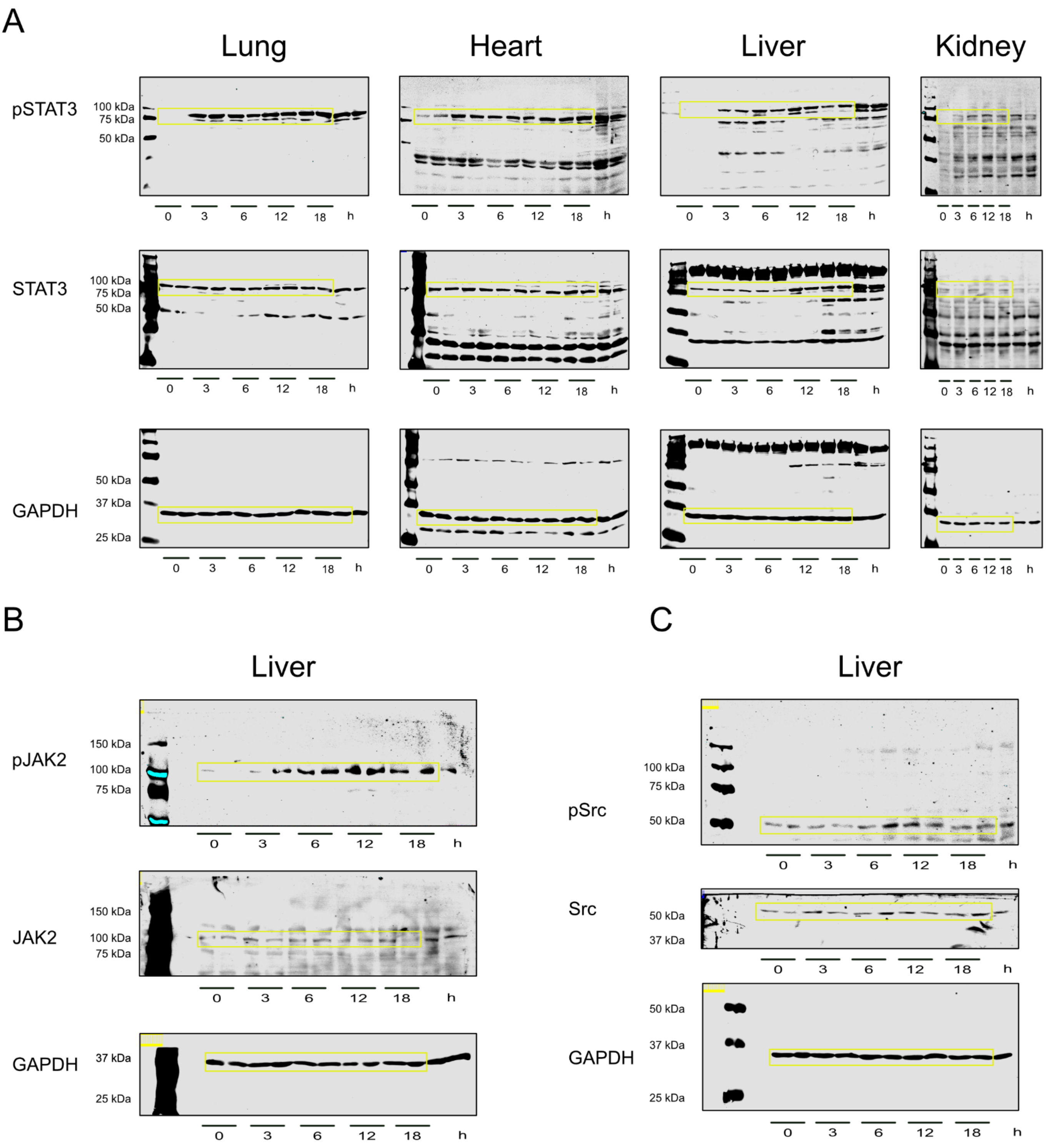

Supplementary Figure S1. Full length Western blot presented in figure 1.

Figure S1 (**A**) The yellow line indicates the cropped regions of pSTAT3, STAT3 and GAPDH in lung, heart, liver, and kidney tissues shown in Fig. 1A. (**B**) The yellow line indicates the cropped regions of pJAK2, JAK2 and GAPDH in liver tissues shown in Fig. 1C. (**C**) The yellow line indicates the cropped regions of pSrc, Src and GAPDH in liver tissues shown in Fig. 1D.

Fig. S2

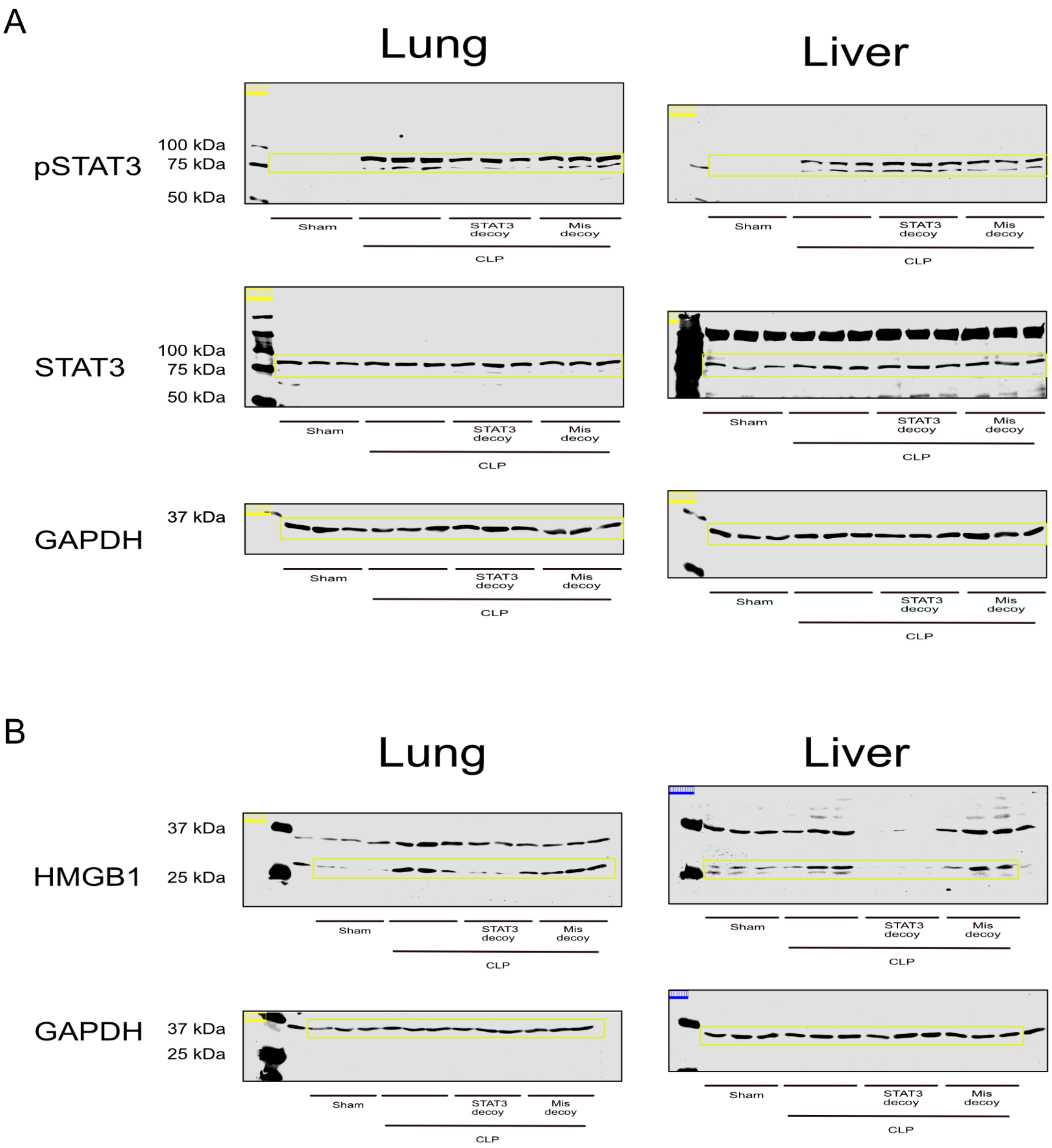

Supplementary Figure S2. Full length Western blot presented in figure 2B and figure 4A.

Figure S2 (**A**) The yellow line indicates the cropped regions of pSTAT3, STAT3 and GAPDH in lung and liver tissues shown in Fig. 2B. (**B**) The yellow line indicates the cropped regions of HMGB1 and GAPDH in lung and liver tissues shown in Fig. 4A.

Fig. S3

A

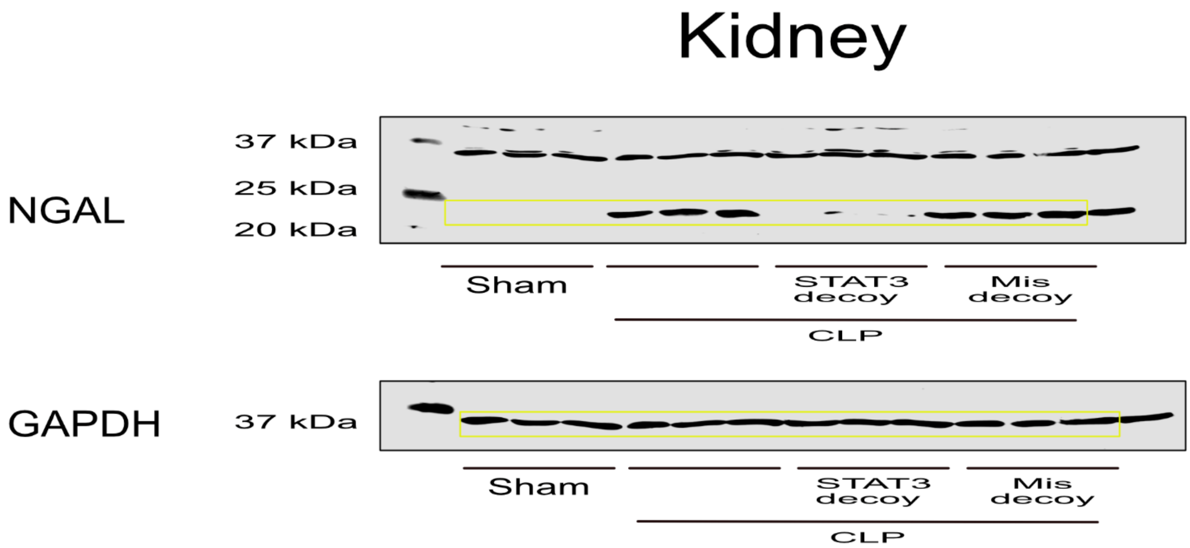

B

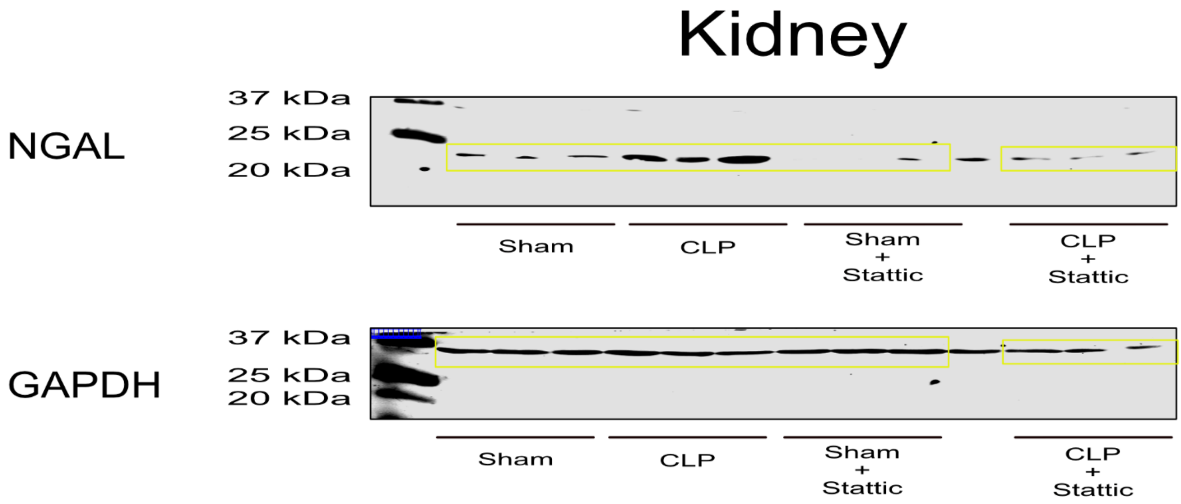

Supplementary Figure S3. Full length Western blot presented in figure 6C and figure 9F.

Figure S3 (**A**) The yellow line indicates the cropped regions of NGAL and GAPDH in kidney tissues shown in Fig. 6C. (**B**) The yellow line indicates the cropped regions of NGAL and GAPDH in kidney tissues shown in Fig. 9F.
